# The vicious cycle of neuroinflammation and ferroptosis in depression: mechanisms and therapeutic targets

**DOI:** 10.3389/fnins.2026.1819436

**Published:** 2026-04-13

**Authors:** Leyi Yao, Ruyu Yan, Chong Sun

**Affiliations:** 1 Queen Mary School, Jiangxi Medical College, Nanchang University, Nanchang, Jiangxi, China; 2 Jiangxi Province Key Laboratory of Brain Science and Brain Health, Department of Neurology, School of Basic Medical Sciences, The Second Affiliated Hospital, Jiangxi Medical College, Institute of Biomedical Innovation, Nanchang University, Nanchang, China

**Keywords:** depression, ferroptosis, neuroinflammation, therapeutic targets, vicious cycle

## Abstract

Depression is one of the leading causes of disability worldwide, yet its underlying mechanisms remain elusive. Both neuroinflammation and ferroptosis are known contributors to the disease, and understanding their interplay is critical for unraveling the pathogenesis of depression. This review explores the relationship between neuroinflammation and ferroptosis in the context of depression. We first delineate the core mechanisms of each process. Subsequently, we focus on the key signaling pathways bridging these processes, including cyclic GMP-AMP synthase (cGAS) stimulator of interferon genes (STING), nuclear factor kappa-B (NF-κB), Janus kinase (JAK)/signal transducer and activator of transcription (STAT) and nuclear factor erythroid 2-related factor 2 (Nrf2), and elucidate how this inflammation-ferroptosis vicious cycle contributes to depression. Finally, we highlight therapeutic agents targeting both processes, suggesting novel treatment directions. By showing that inflammation and ferroptosis form a vicious cycle, this work offers a clearer perspective on the pathogenesis of depression and identifies specific therapeutic targets for breaking this pathological loop.

## 1 Introduction

Depression is a common mental disorder, with a global prevalence that has increased significantly over the past three decades, especially in Asia, North Africa, and the Middle East (Liu et al., 2020). Depressive disorders rank as the third most prevalent mental disorders worldwide (GBD 2019 Mental Disorders Collaborators, 2022). However, the clinical utility of conventional antidepressants is often limited by modest efficacy and a range of adverse effects (Cowen, 2008; Pigott et al., 2010; Strawn et al., 2023; Zisook et al., 2006). For instance, selective serotonin reuptake inhibitors (SSRIs), the most commonly prescribed antidepressants, carry a black-box warning for increased suicidal risk, and frequently cause sexual dysfunction in up to 80% of female patients (Fornaro et al., 2019; de Aquino et al., 2025). Besides, most antidepressants share common challenges, including a marked therapeutic delay of several weeks, limited bioavailability, poor blood–brain barrier permeability, and instability in the gastrointestinal tract (Ferrari and Villa, 2017; Văruț et al., 2025). Moreover, approximately one-third of patients exhibit treatment-resistant depression (TRD) and fail to respond to existing pharmacotherapies, while some patients, particularly children and adolescents, may experience spontaneous improvement even without treatment (Richardson et al., 2025; Kennard et al., 2009). These observations collectively suggest that the underlying pathophysiology of depression needs to be further investigated. Thus, elucidating the mechanisms of depression and developing novel therapeutics with distinct mechanisms of action remain a critical priority.

While the exact etiology of depression is still under investigation, inflammation has emerged as a significant factor in its development (Barton, 2008). Stress can induce inflammation, which in turn promotes depressive-like behaviors in some patients (Bierhaus et al., 2003; Irwin and Cole, 2011; Kiecolt-Glaser et al., 2015; Shelton and Miller, 2010). Furthermore, anti-inflammatory treatments can alleviate depressive-like behaviors, indicating the potential antidepressant effects of anti-inflammatory drugs (Kang et al., 2011; Liu H. et al., 2023; Guo et al., 2019). Meanwhile, ferroptosis—an iron-dependent programmed cell death (PCD) first reported by Dixon et al.—has emerged as a key player in depression, with studies documenting its presence in neurons and glia and showing that suppressing it alleviates depressive-like behaviors (Dang et al., 2022; Shen et al., 2024b; Mao et al., 2024; Li E. et al., 2023). Emerging evidence suggests that these two pathological processes are not independent; rather, they engage in a self-amplifying vicious cycle—inflammation potentiates ferroptosis, and ferroptotic products and intracellular iron overload in turn fuel neuroinflammation (Li E. et al., 2023; Xin et al., 2020; Yu et al., 2020; Wang et al., 2019; Li et al., 2019). Elucidating this vicious cycle is fundamental to understanding depression pathogenesis and to identifying new therapeutic targets.

This review begins by delineating the core mechanisms of neuroinflammation and ferroptosis individually and then proceeds to explore their molecular interplay within the central nervous system (CNS). By framing these processes as a vicious cycle, we aimed to provide an integrated perspective on how neuroinflammation and ferroptosis collectively drive depressive pathology. Finally, we reported therapeutic agents that target this vicious cycle, thereby providing novel therapeutic strategies for depression.

## 2 Neuroinflammation in depressive disorder: multidimensional pathogenic mechanisms

**Figure 1:**
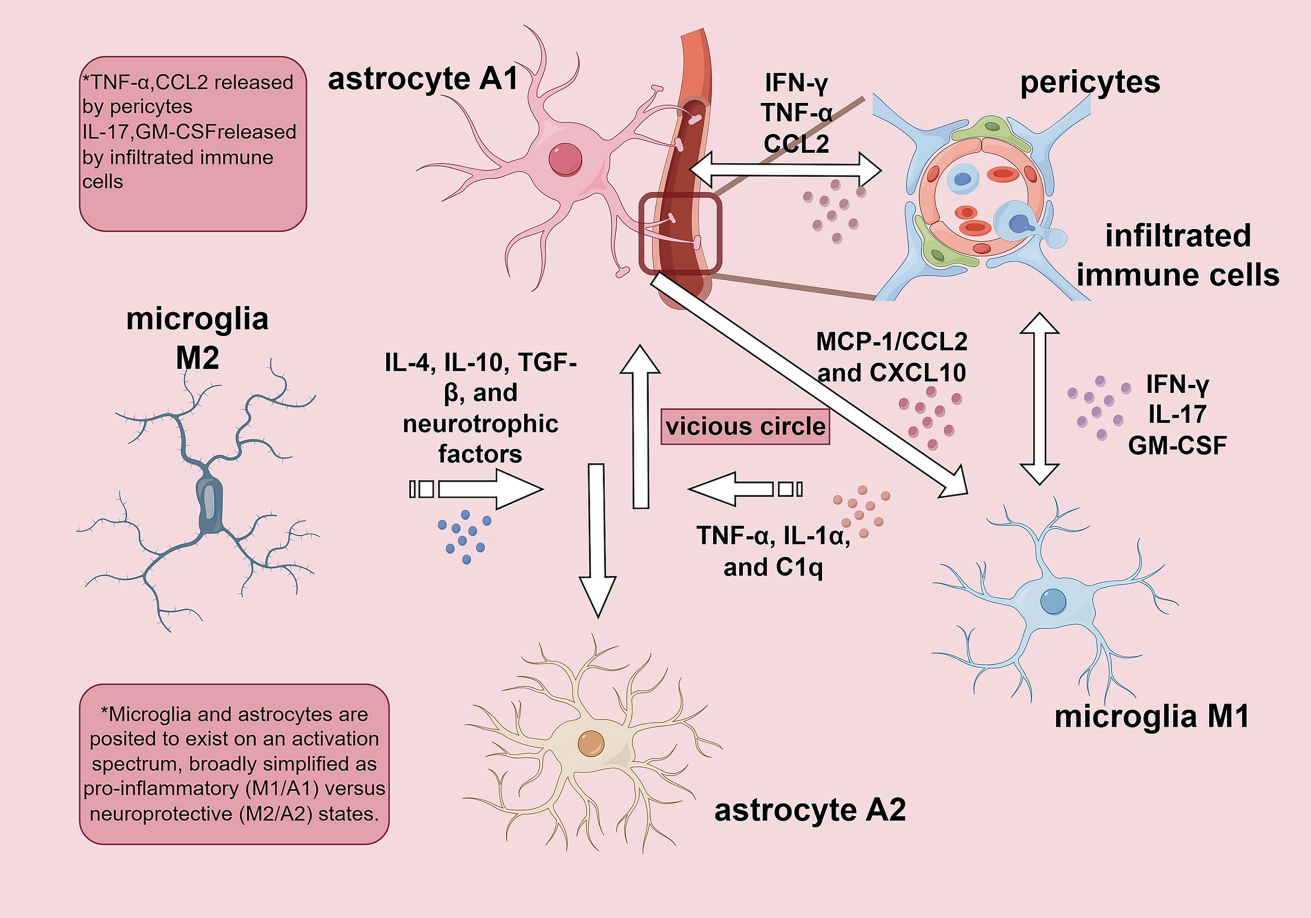
Interactive network of glial and infiltrated immune cells in central neuroinflammation in depression. This schematic illustrates the central cellular interactions that drive sustained neuroinflammation within the central nervous system (CNS) in depression. At the core of this network is a self-perpetuating, pathological cycle: Activated “M1 microglia” release pro-inflammatory cytokines such as tumor necrosis factor-alpha (TNF-α). These cytokines push astrocytes toward a neurotoxic “A1 astrocyte” phenotype. In turn, A1 astrocytes secrete additional inflammatory mediators, including chemokines like CCL2 and CXCL10, which further activate microglia, closing a positive feedback loop. This cycle is amplified by the recruitment of “peripheral immune cells”—for example, IL-17-producing T cells—across the blood–brain barrier. “Pericytes,” which surround blood vessels in the CNS, also adopt a pro-inflammatory profile, releasing TNF-α and CCL2, thereby enhancing both immune cell infiltration and glial activation. Opposing this pro-inflammatory cascade is a resolution pathway driven by mediators such as IL-4, IL-10, and transforming growth factor-beta (TGF-β). These signals promote protective “M2 microglia” and “A2 astrocyte” phenotypes, which support tissue repair and suppress inflammation. However, in major depressive disorder, this reparative axis is often overwhelmed. The persistent dominance of M1/A1 activation and peripheral immune infiltration leads to sustained neuroinflammation, which is thought to contribute to maladaptive behavioral responses, including anhedonia, psychomotor retardation, and mood dysregulation—core features of depression. Home for Researchers. (2022). FigDraw(Version 2.0) [Computer software]. https://www.figdraw.com/

Neuroinflammation is an evolutionarily conserved defense mechanism in the CNS, triggered by insults such as infection or injury and mediated through the coordinated activation of resident glial cells and recruited immune cells (Kopp et al., 2022). These cells release proinflammatory mediators including cytokines, chemokines, and reactive oxygen species to clear threats and restore tissue homeostasis. While acute neuroinflammation is essential for CNS protection, its persistent dysregulation transforms into a pathogenic force that drives neuronal damage and underpins a broad spectrum of neurological and psychiatric conditions. Critically, this maladaptive neuroinflammatory state has been increasingly implicated in the pathophysiology of major depressive disorder (MDD) (Tastan and Heneka, 2024; Sălcudean et al., 2025). This link is thought to arise from prolonged immune activation, which disrupts several brain processes central to mood regulation. This activation is characterized by the sustained activation of neuroglial cells, such as microglia and astrocytes (Yang et al., 2020). Therefore, the dynamic phenotypic shift of glial cells, particularly the interplay between microglia and astrocytes, is emphasized as a central mechanism of neuroinflammation (Figure 1).

Under physiological conditions, microglia maintain central nervous system (CNS) homeostasis through a dynamic and context-dependent spectrum of functional states, traditionally described as a balance between pro-inflammatory and protective responses (Tastan and Heneka, 2024). In neuropsychiatric disorders as depression, this balance persistently shifts toward a pro-inflammatory phenotype. Damage-associated molecular patterns, such as high mobility group box 1 (HMGB1), initiate this phenotypic shift by engaging signaling pathways like toll-like receptor 4 (TLR4) and the receptor for advanced glycation end products (RAGE), which in turn activate downstream effectors, including nuclear factor kappa-B (NF-κB) and the NOD-like receptor thermal protein domain associated protein 3 (NLRP3) inflammasome (Tastan and Heneka, 2024; Afridi and Suk, 2023; Ajoolabady et al., 2025). Concurrently, impaired function of regulatory receptors, such as triggering receptor expressed on myeloid cells 2 (TREM2), reduces phagocytic clearance and attenuates anti-inflammatory signaling, thereby impairing endogenous resolution pathways (Ajoolabady et al., 2025). Upon activation, microglia drive neuroinflammation through the release of mediators, including tumor necrosis factor-alpha (TNF-α), interleukin-1 alpha (IL-1α), and complement component 1q (C1q), which promote the conversion of astrocytes into a neurotoxic A1-reactive phenotype (Tastan and Heneka, 2024). Together with pericytes and infiltrating peripheral immune cells, these cells sustain a chronic pro-inflammatory environment characterized by elevated cytokines (e.g., interleukin-1 beta [IL-1β], TNF-α, interleukin-6 [IL-6]), complement system activation (e.g., C1q, complement [C3]), and persistent inflammasome activity. These cascades collectively drive synaptic loss, axonal damage, dysregulated calcium signaling, impaired long-term potentiation, and altered neural network connectivity (Ajoolabady et al., 2025). While useful as a simplified framework that characterizes microglia as either pro-inflammatory (M1) or protective/repair-oriented (M2), the classical M1/M2 dichotomy does not fully capture the diverse and dynamic activation states of microglia, which often exist along a phenotypic continuum between pro-inflammatory and protective functions (Tastan and Heneka, 2024; Yang et al., 2020). The resulting structural and functional impairments in neuronal integrity contribute to cognitive deficits, mood dysregulation, and other neuropsychiatric symptoms, thereby reinforcing a self-perpetuating cycle of neuroimmune dysregulation.

Astrocytes are now recognized as active regulators of central nervous system (CNS) homeostasis and neuroinflammation (Giovannoni and Quintana, 2020). Under physiological conditions, they contribute to blood–brain barrier (BBB) integrity, ion and fluid balance through aquaporin-4 (AQP-4) channels, synaptic modulation via glutamate transporters (excitatory amino acid transporters [EAATs], including glutamate transporter-1 [GLT-1] and glutamate–aspartate transporter [GLAST]), and metabolic support through lactate shuttling (Yang et al., 2020). In neuroinflammatory contexts, astrocytes undergo reactive transformation, acquiring phenotypic states that may exert either detrimental or protective effects, depending on microenvironmental signals. Microglia play a crucial role in orchestrating this phenotypic shift: upon activation, they release inflammatory mediators such as IL-1α, TNF-α and C1q, which induce a pro-inflammatory reactive astrocyte phenotype (often termed “A1-like” or neurotoxic reactive state) (Zhao et al., 2024; Lee et al., 2023; Jha et al., 2019). Such reactive astrocytes are characterized by reduced glutamate uptake capacity and increased release of pro-inflammatory cytokines, including TNF-α and IL-1β, which contribute to synaptic dysfunction and neuronal injury. In contrast, under reparative conditions, microglia may support an alternative reactive astrocyte phenotype (“A2-like” or neuroprotective state), associated with the secretion of anti-inflammatory mediators such as interleukin-10 (IL-10), interleukin-4 (IL-4), transforming growth factor-β (TGF-β), and neurotrophic factors (Zhao et al., 2024). This phenotypic transition reflects a bidirectional crosstalk: activated astrocytes, in turn, modulate microglial activity through chemokines such as monocyte chemoattractant protein-1 (MCP-1/CCL2) and C-X-C motif chemokine ligand 10 (CXCL10), thereby amplifying or attenuating neuroinflammatory cascades (Yang et al., 2020). Thus, the dynamic interaction between microglia and astrocytes, mediated by specific cytokine and chemokine signaling pathways, critically determines neuroinflammatory trajectories and influences both degenerative and regenerative processes in CNS disorders (Jha et al., 2019; Liu et al., 2020).

Moreover, pericytes and infiltrating peripheral immune cells also contribute to the neuroinflammatory cascade. They influence the initiation, progression, and resolution of neuroinflammation through multiple mechanisms, including the release of inflammatory mediators, regulation of immune cell infiltration, interactions with microglia and endothelial cells, inflammasome activation, extracellular matrix degradation, and phenotypic plasticity (Tastan and Heneka, 2024; Liu et al., 2020). In summary, for patients with depression, neuroinflammation—primarily driven by the chronic activation of glial cells—induces a cascade of structural and functional alterations across key emotion- and cognition-regulating brain regions (Sălcudean et al., 2025; Troubat et al., 2021). For example, it leads to reduced hippocampal volume and impaired neurogenesis; prefrontal cortical gray matter atrophy and hypoconnectivity; hyperactivity of the amygdala and heightened connectivity; structural alterations and dysfunctional conflict processing in the anterior cingulate cortex; and dysregulated dopaminergic signaling and blunted reward response in the nucleus accumbens (Sălcudean et al., 2025). These coordinated damages across multiple brain regions collectively disrupt the neural circuits governing emotion regulation, cognitive execution, and motivational reward, ultimately resulting in the core clinical symptoms of persistent low mood, anhedonia, executive dysfunction, and cognitive impairment in patients (Troubat et al., 2021; Reyes-Martínez et al., 2023). Importantly, these neuroinflammatory damages may be further aggravated by a vicious cycle with ferroptosis, as discussed in the later sections.

In depression, neuroinflammation is induced by psychosocial stress through various mechanisms including activation of microglia, activation of the sympathetic nervous system (SNS), and glucocorticoid resistance (Miller and Raison, 2016; Amit et al., 2009; Berkenbosch et al., 1987; Miller et al., 2002). The activation of microglia directly results in the secretion of proinflammatory cytokines in the CNS, which may attract peripheral monocytes to further promote neuroinflammation (Miller and Raison, 2016; Tang and Le, 2016). On the other hand, activation of the SNS in turn activates peripheral leukocytes to promote expression of NF-κB-mediated pro-inflammatory immune response genes such as IL-1β, IL-6, and TNF-α via adrenaline and noradrenaline, which may generate an inflammatory environment in the periphery (Amit et al., 2009; Cole et al., 2010; Grebe et al., 2010). Furthermore, glucocorticoid resistance reduces the anti-inflammatory effects of glucocorticoids, which aggravates inflammation (Berkenbosch et al., 1987; Miller et al., 2002; Sapolsky et al., 1987; Besedovsky et al., 1986; Duman and Aghajanian, 2012). Finally, the peripheral inflammatory cytokines can not only cross the BBB but also bind to receptors on endothelial cells to activate the production of prostaglandins (PGs), which promotes neuroinflammation (Schiepers et al., 2005; Friedman, 2001; Utsuyama and Hirokawa, 2002; Watkins et al., 1995). Thus, neuroinflammation can be established under chronic social-environmental threats.

Inflammatory cytokines affect neurotransmission through several pathways, a process which is related to neuronal dysfunction and synaptic plasticity in depression. For example, inflammatory cytokines can induce the oxidative loss and decrease the concentration of tetrahydrobiopterin (BH4) (Felger et al., 2013; Werner et al., 2003). As an enzymatic cofactor in the synthesis of monoamine neurotransmitters, including serotonin and dopamine, reduced BH4 levels contribute to decreased 5-Hydroxytryptamine (5-HT) and dopamine (DA) levels, which induces depressive-like behaviors (Nasser et al., 2014; Kwak et al., 2013; Zeng et al., 2004; Homma et al., 2011). Furthermore, inflammatory cytokines induce the activation of indoleamine 2,3-dioxygenase (IDO), an enzyme that catalyzes the production of kynurenine from tryptophan, thereby reducing the alternative metabolic pathway that yields 5-HT (Raison et al., 2010; Maes et al., 2011). Furthermore, activation of the kynurenine pathway promotes the production of quinolinic acid, an excitotoxic compound that promotes glutamate release and blocks glutamate reuptake (Steiner et al., 2011; Tavares et al., 2002). Besides, inflammatory cytokines also directly affect extracellular levels of glutamate by downregulating the expression of glutamate reuptake pump on astrocytes and promoting glutamate release of astrocytes (Tilleux and Hermans, 2007). Excessive glutamate over activates the postsynaptic glutamate receptors, disrupting intracellular calcium homeostasis and contributing to in neuronal death (Simões et al., 2018; Heath and Shaw, 2002; Scheefhals and MacGillavry, 2018). Furthermore, overactivation of N-Methyl-D-aspartic acid (NMDA) receptors by glutamate reduces brain-derived neurotrophic factor (BDNF) production, resulting in decreased neurogenesis. Therefore, neuroinflammation induced in depression disrupts neurotransmission and thus promotes the development of depression (Hardingham et al., 2002; Koo et al., 2010; Goshen et al., 2008).

Neuroinflammation plays a critical role in hippocampus atrophy as well (Zhang et al., 2024; Bajrami et al., 2022; Cherbuin et al., 2019; Semmler et al., 2013). On the one hand, inflammation reduces adult hippocampal neurogenesis (Borsini et al., 2021; Yirmiya and Goshen, 2011; Chen P. et al., 2023). On the other hand, inflammation induces PCD of various cells in the hippocampus and prefrontal cortex (PFC), including neuron, glial cells, and neural stem cells (NSCs) (Li W. et al., 2023; Li et al., 2026; Nikolopoulos et al., 2023). As the hippocampus and adult hippocampal neurogenesis (AHN)—a neuronal maturation process dependent on NSCs—are important for learning, cognition, and memory, disrupted hippocampal function and suppressed neurogenesis contribute to the development of depression (Yirmiya and Goshen, 2011; Hitti and Siegelbaum, 2014; Time, 2003; Tartt et al., 2022; Ballesteros et al., 2025). Notably, hippocampal atrophy and impaired neurogenesis are not only consequences of neuroinflammation but also sites where ferroptosis can be triggered, potentially establishing a local vicious cycle that exacerbates structural and functional deficits.

## 3 Ferroptosis: a novel player in depression pathogenesis

Dixon and his team introduced the concept of ferroptosis in 2012, characterizing it as an iron-dependent mode of programmed cell death marked by the build-up of lipid hydroperoxides (Dixon et al., 2012). The morphological features of ferroptosis primarily involve mitochondrial alterations including rupture of the mitochondrial membrane, reduced mitochondrial size with increased mitochondrial membrane densities, and loss of mitochondrial cristae (Li J. et al., 2020). In addition, disruption of plasma membrane integrity occurs in cells undergoing ferroptosis, as evidenced by propidium iodide (PI) staining (Liu et al., 2020). Nevertheless, the nucleus of ferroptotic cells maintains basic structural integrity (GBD 2019 Mental Disorders Collaborators, 2022).

Iron homeostasis and its dysregulation are the core of ferroptosis. First, transmembrane iron transportation is a key regulator of intracellular iron homeostasis. Fe^3+^ is bond by transferrin (TF) in the plasma and combines with transferrin receptor protein 1 (TFR1) on the cell membrane, facilitating cellular entry via endocytosis. Protons are pumped into the endosome, lowering the pH and triggering the release of Fe^3+^ from the TF-TFR1 complex. The released Fe^3+^ is then reduced to Fe^2+^ by six-transmembrane epithelial antigen of prostate 3 (STEAP3) and exported from the endosome into the cytoplasm by divalent metal transporter1 (DMT1). Following iron release, the TF-TFR1 complex is recycled back to the cell membrane via the endosome, which recycles TF and TFR1 (Candelaria et al., 2021). Second, cytoplasmic Fe^2+^ can be exported from cells by ferroportin (FPN), a process inhibited by hepcidin, which blocks and ubiquitinates FPN. Intracellular Fe^2+^ serves dual roles: it can be securely stored via ferritin binding in the cytoplasm or mitochondria, or it can function as a cofactor in the synthesis of heme and iron–sulfur clusters (Kowdley et al., 2021; Richardson et al., 2010). However, these intracellular iron storage mechanisms are reversible. For example, cytosolic ferritin can release Fe^2+^ through a process mediated by nuclear receptor coactivator 4 (NCOA4)-induced autophagy, known as ferritinophagy (Gao et al., 2016). Moreover, although the autophagy of mitochondria (mitophagy) may sequester iron in mitophagosomes to prevent iron from releasing into the cytoplasm in the early stages of iron overload, extensive mitophagy can conversely trigger Fe^2+^ liberation, elevate cytosolic Fe^2+^ levels, and amplify ferroptosis (Yu et al., 2022; Rademaker et al., 2022).

In the presence of Fe^2+^, ferroptosis is primarily induced through two pathways: the Fenton reaction-dependent pathway and the Fe^2+^-dependent enzymatic pathway (Murphy, 2009; Yang et al., 2016). Both pathways utilize polyunsaturated fatty acid (PUFA)-containing phospholipids (PLs) as substrates. PUFA-PLs are synthesized from PUFA through the sequential action of acyl-coenzyme A (CoA) synthetase long-chain family member 4 (ACSL4) and lysophosphatidylcholine acyltransferase3 (LPCAT3). These PUFA-PLs can then react with hydroxyl radicals generated by the Fenton reaction (Fe^2+^ reacting with hydrogen peroxide [H_2_O_2_]; H_2_O_2_ is primarily derived from the superoxide leaking from mitochondrial electron transport chain [ETC] complex I and III) to form PUFA radicals (PUFA**·**). PUFA**·** subsequently reacts with oxygen to yield PUFA peroxyl radicals (PUFA-OO·) and, ultimately, phospholipid hydroperoxides (PL-OOH) (Yang et al., 2016). Alternatively, PUFA-PLs can directly react with oxygen to yield PL-OOH in a process catalyzed by Fe^2+^ and lipid-peroxidizing enzymes such as lipoxygenases [ALOXs] and cytochrome P450 oxidoreductase [POR] (Yang et al., 2016; Koppula et al., 2021).

Glutathione peroxidase 4 (GPX4) is an essential peroxidase that suppresses ferroptosis, requiring glutathione (GSH) as a cofactor to catalyze the reduction of PL-OOH. GSH synthesis requires cystine, which is imported by cystine/glutamate antiporter (system X_C_^−^), a system composed of solute carrier family 7 member 11 (SLC7A11) and solute carrier family 3 member 2 (SLC3A2)(Dixon et al., 2012). Another system that suppresses ferroptosis, FSP1-CoQ_10_-NAD(P)H, operates in parallel to the GSH/GPX4 pathway. Ferroptosis suppressor protein 1 (FSP1) reduces coenzyme Q10 (CoQ) by utilizing nicotinamide adenine dinucleotide phosphate hydrogen (NADPH) to generate CoQH_2_, a radical-trapping antioxidant (RTA) that traps lipid peroxyl radicals and thus inhibits ferroptosis (Doll et al., 2019; Bersuker et al., 2019).

Emerging evidence implicates ferroptosis in the pathogenesis of depression (Dang et al., 2022; Shen et al., 2024b). For instance, hippocampal ferroptosis and depressive-like behaviors were observed in lipopolysaccharides (LPS)-induced mice, whereas treatment with ferrostatin-1 (Fer-1), a ferroptosis inhibitor, alleviated depressive-like behaviors of chronic unpredictable mild stress model (CUMS)-induced mice (Mao et al., 2024; Li E. et al., 2023). In addition, ferroptosis contributes to dysfunction of various CNS cell types in depression. Therefore, ferroptosis and neuroinflammation are not parallel pathological features; they are mechanistically coupled, forming a bidirectional vicious cycle that we will dissect in the following sections.

## 4 Bidirectional crosstalk between neuroinflammation and ferroptosis in depression

**Figure 2:**
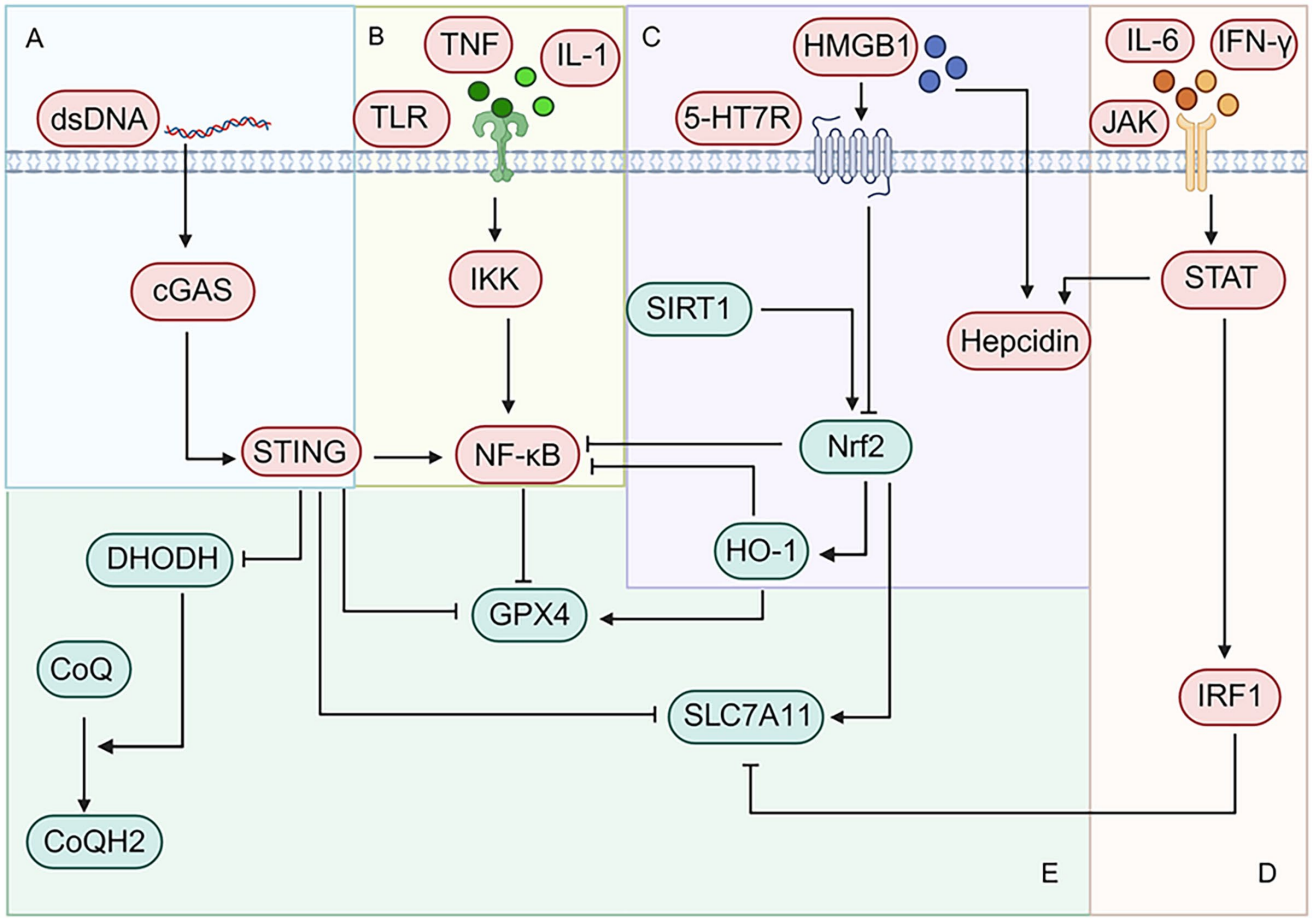
Main pathways linking ferroptosis and inflammation in the central nervous system. **(A)** The cyclic GMP-AMP synthase (cGAS)/stimulator of interferon genes (STING) pathway is activated by damage-associated molecular patterns (DAMPs) such as double-stranded DNA (dsDNA). STING activates nuclear factor kappa-B (NF-κB), downregulates dihydroorotate dehydrogenase (DHODH), glutathione peroxidase 4 (GPX4), and solute carrier family 7 member 11 (SLC7A11). Activation of NF-κB promoting further production of proinflammatory cytokines and suppression of anti-ferroptotic genes. DHODH is a mitochondrial enzyme that suppresses lipid peroxidation and ferroptosis. GPX4 catalyzes the reduction of PL-OOH, which needs cystine/glutamate antiporter (system X_C_^−^) (a system composed of SLC7A11 and solute carrier family 3 member 2) to transport the ingredients for its cofactor. STING thus promotes neuronal ferroptosis via inhibiting DHODH and GPX4 system. **(B)** Inflammatory cytokines combine to TLR to activate IKK and in turn NF-κB, which not only promotes inflammation but also downregulates GPX4. **(C)** Nuclear factor erythroid 2-related factor 2 (Nrf2)/ heme oxygenase-1 (HO-1) axis not only inhibits NF-κB to supress inflammation but also upregulates GPX4 and SLC7A11 to reduce phospholipid hydroperoxides (PL-OOH) and prevent ferroptosis. However, this axis was reported to be inhibited by HMGB1-Serotonin receptor 7 (5HT7R) axis, resulting ferroptosis of M2 microglia. In contrast, edaravone (EDV) activates Nrf2/HO-1 axis via SIRT1, which alleviates neuronal ferroptosis. Besides, HMGB1 activates the upregulation of hepcidin via various pathways. Since hepcidin inhibits Fe^2+^ export, HMGB1 is a critical target for intracellular iron accumulation. **(D)** The Janus kinase (JAK)/signal transducer and activator of transcription (STAT) is activated by inflammatory cytokines, which in turn upregulates hepcidin and interferon regulatory factor 1 (IRF1). IRF1 downregulates SLC7A11, inhibiting GPX4-dependent anti-ferroptotic pathway. **(E)** The core anti-ferroptotic targets of discussed pathways (cGAS/STING, NF-κB, Nrf2/HO-1 and JAK/STAT) are DHODH, GPX4, and SLC7A11. DHODH reduces CoQ to CoQH_2_. CoQH_2_ serves as a potent radical-trapping antioxidant, directly capturing and removing lipid peroxyl radicals on the inner mitochondrial membrane. This pathway runs in parallel with GPX4, offering another line of defense against ferroptosis. The GPX4-dependent anti-ferroptotic pathway and the DHODH-mediated PLOOH clearance pathway in mitochondria are the main characters in this vicious cycle. Created in BioRender. yao, L. (2026) https://BioRender.com/bhfx1mb

Although the individual roles of neuroinflammation and ferroptosis in the development of depression have received considerable attention, the bidirectional crosstalk between these processes and their specific molecular pathways remains relatively unexplored. By understanding these connections, the complex mechanism of depression pathogenesis can be further elucidated, thereby facilitating the development of novel antidepressant therapies that target both processes. The crosstalk between inflammation and ferroptosis has been systematically reviewed, however, its specific role in the CNS, especially in depression, has not been thoroughly addressed. This section focuses on the molecular wiring that sustains the vicious cycle between neuroinflammation and ferroptosis. By mapping how inflammatory pathways promote ferroptosis and how ferroptotic signals re-ignite inflammation, we can identify the leverage points to break the cycle (Figure 2).

### 4.1 Key molecular pathways related to neuroinflammation-mediated ferroptosis

#### 4.1.1 Cyclic GMP-AMP synthase/stimulator of interferon genes

The stimulator of interferon genes (STING) pathway is activated by double-stranded DNA (dsDNA), which activates the expression of interferon (IFN) via interferon regulatory factor 3 (IRF3) (Liu et al., 2015). Beyond its role in promoting inflammation, STING has also been reported to promote ferroptosis in neurons and microglia, mainly via two mechanisms: inhibition of DHODH and suppression of GPX4.

The level of dihydroorotate dehydrogenase (DHODH) was significantly reduced in neurons treated with oxyhemoglobin, a reduction mediated by the CGAS/STING pathway (You et al., 2025). DHODH, a mitochondrial enzyme that suppresses lipid peroxidation in mitochondria and ferroptosis via CoQ (Cao et al., 2025; Mao et al., 2021). The CGAS/STING/DHODH pathway plays a role in not only SAH-induced neuronal ferroptosis but also chronic restraint stress (CRS)-induced hippocampal ferroptosis, resulting in cognitive impairments (You et al., 2025; Zhang J. B. et al., 2025). Inhibition of the CGAS/STING activation and upregulation of DHODH have been shown to reverse depressive-like behaviors, including cognitive and memory defects (You et al., 2025; Zhang J. B. et al., 2025; Tian et al., 2023). Compared with DHODH, the suppression of GPX4 by CGAS/STING has been more well-documented. In a study related to multiple sclerosis (MS), prolonged exposure of neurons to interferon-γ (IFN-γ) induced expression and activation of STING. Rather than canonically activating downstream proinflammatory cytokines, STING promotes autophagic degradation of GPX4, thereby triggering neuronal ferroptosis (Woo et al., 2024). Furthermore, inhibiting the cGAS/STING pathway with RU.521 upregulates the expression of SLC7A11 and GPX4, which mitigates microglial ferroptosis (Sui et al., 2025). Given that both CGAS/STING activation and reduced GPX4 levels have been observed in various neural cells in depression models, their interaction may contribute to ferroptosis and pathological injury in the hippocampus and PFC (Liu et al., 2022; Yuan et al., 2025; Duan et al., 2022; Du et al., 2025).

While the CGAS/STING pathway regulates neuronal and microglial ferroptosis, direct evidence of this connection in depression remains limited. Nevertheless, this regulatory relationship has been demonstrated in various tissues and diseases, suggesting that the mechanisms by which cGAS/STING promotes ferroptosis may be conserved across different cell types and pathological contexts (Gao et al., 2023; Wu et al., 2022; Zhu et al., 2024). Therefore, CGAS/STING may contribute to ferroptosis in depression, given its involvement in multiple CNS disorders, its capacity to induce ferroptosis in various central and peripheral tissues, and importantly, the observation that its inhibition can effectively alleviate depressive-like behaviors. The cGAS/STING pathway thus serves as a critical amplifier of the vicious cycle, linking DNA damage-triggered inflammation to ferroptosis execution.

#### 4.1.2 JAK/STAT

Janus kinase (JAK)/signal transducer and activator of transcription (STAT) is activated by extracellular cytokines such as IL-6 and IFN-γ, and plays a key role in promoting inflammation (Xin et al., 2020). In addition to its inflammatory function, this pathway also promotes ferroptosis, mainly through two downstream effectors: hepcidin and IRF1.

Hepcidin, which can be expressed by CNS cells and transported from the liver, is a downstream target of the JAK–STAT (Zechel et al., 2006; Raha-Chowdhury et al., 2015; Qian et al., 2014). Hepcidin promotes intracellular iron overload via inhibiting iron export through ferroportin blockage and ubiquitination (Kowdley et al., 2021). In the nervous system, the IL-6/JAK/STAT pathway triggers hepcidin expression, as demonstrated in astrocytes after hemoglobin treatment and in neurons via microglial IL-6 release (Qian et al., 2014; Xiong et al., 2016). While some studies have reported anti-inflammatory effects of hepcidin on astrocytes, in other cell types, especially neurons, hepcidin promotes ferroptosis via blocking FPN (Kowdley et al., 2021; Urrutia et al., 2017; Davaanyam et al., 2023), resulting in neuronal loss and contributing to depression (Zhang N. et al., 2025; Farajdokht et al., 2015). The transcription of interferon regulatory factor 1 (IRF1), another downstream factor activated by JAK–STAT, inhibits GPX4 activity by downregulating SLC7A11 (Li et al., 2014; Blaszczyk et al., 2016; Feng et al., 2025; Kong et al., 2021), a component of system X_C_^−^ that is essential for GSH synthesis (Dixon et al., 2012). Suppressing IRF1 expression with cynaroside, thereby promoting SLC7A11 expression, alleviated ferroptosis in LPS-induced BV-2 cells and reduced hippocampal inflammation and depressive-like behaviors (Zhou Y. et al., 2024). Together, the JAK/STAT/hepcidin and JAK/STAT/IRF1 pathways promote the loss of neurons and microglia, thereby contributing to brain damage and depressive-like behaviors.

#### 4.1.3 NF-κB

The NF-κB pathway can be activated by the binding of Toll-like receptors to ligands such as TNF and IL-1, serving as a master regulator of inflammation (Yu et al., 2020). Although NF-κB is well known for promoting inflammation, it also modulates ferroptosis by suppressing GPX4 and regulating LCN2 expression.

The level of GPX4 is regulated by NF-κB. During ischemic stress, activated NF-κB downregulates GPX4 and system X_C_^−^ while upregulating TFR1, thereby promoting microglial ferroptosis (Zheng et al., 2024; García-Weber and Arrieumerlou, 2021). Another study related to CIRI also supported the inhibitory effects of NF-κB on GPX4 (Xie et al., 2025). In depression, while direct evidence linking NF-κB and GPX4 remains limited, it has been established that both NF-κB activation and GPX4 suppression contribute to brain injury, including neuronal loss, microglial overactivation, and reduced adult hippocampal neurogenesis (AHN) (Li et al., 2021; Erady et al., 2025).

However, LCN2, a downstream target of NF-κB, exhibits paradoxical effects on ferroptosis—both promoting and inhibiting it in different contexts—adding complexity to the role of NF-κB in this process. LCN2, an acute phase protein secreted by activated astrocytes or neutrophils, has been shown to promote neuronal ferroptosis via downregulation of SLC7A11 in various CNS disease models, including epilepsy and diabetes-associated ischemic stroke (Zhou Z. et al., 2024; Wang et al., 2024). However, NF-κB/LCN2 pathway was reported to inhibit ferroptosis of neurons, which partially protected TBI-induced mice from brain damage (Wang et al., 2025). Although the role of LCN2 in downregulating SLC7A11 has not been extensively studied in depression, LCN2 upregulation and SLC7A11 downregulation were observed in streptozotocin (HFD-STZ)-induced mice, contributing to neuronal loss and cognitive dysfunction (Xie et al., 2023). Furthermore, neuronal ferroptosis and depressive-like behaviors could be elevated by targeting LCN2(Xie et al., 2023; Chen Y. et al., 2023; Yang et al., 2023).

### 4.2 Key molecular pathways related to ferroptosis-induced neuroinflammation

Ferroptosis not only is a consequence of neuroinflammation but also actively propagates the vicious cycle through the release of damage-associated molecular patterns (DAMPs) and glial activation. DAMPs released from ferroptotic cells—such as lipid oxidation products [hydroxynonenal (4-HNE) and oxidized phospholipids (oxPLs)], HMGB1, and dsDNA—activate the TLR4-MyD88-NF-κB and cGAS-STING pathways, driving the production of proinflammatory cytokines and type I interferons (Wang et al., 2019; Liu et al., 2015; Feng et al., 2024; Imai et al., 2008; Dvorkin et al., 2024). These sequences—from ferroptosis to inflammation mediated by released DAMPs—explain the ferroptosis arm of the vicious cycle. Once initiated, it primes an inflammatory microenvironment for further ferroptotic events. Concurrently, glial cells activated by ferroptosis further amplify inflammation; for instance, iron accumulation in microglia and astrocytes promotes the release of proinflammatory cytokines, exacerbating neuroinflammation and neuronal damage (Ryan et al., 2023; Moro et al., 2025) Through their collective effects, ferroptosis of CNS cells further promotes neuroinflammation.

### 4.3 Shared molecular pathways

The following contents summarize several key pathways that affect both neuroinflammation and ferroptosis simultaneously.

#### 4.3.1 Nrf2

Nuclear factor erythroid 2 (Nrf2), a protein that regulates genes involved in both ferroptosis and inflammatory pathways, represents a target in depression. Under homeostatic conditions, Kelch-like ECH-associated protein 1 (Keap1) binds to Nrf2 to sequester Nrf2 in the cytoplasm, promoting its degradation via the proteasome (Itoh et al., 1999). Upon stimulation, Nrf2 is released, translocates to the nucleus, and promotes the expression of antioxidant genes, including *heme oxygenase 1* (*HMOX1*), *NAD(P)H quinone dehydrogenase 1* (*NQO1*), *superoxide dismutase* (*SOD*), and *catalase* (*CAT*) (Kansanen et al., 2013; He et al., 2020). *HMOX1* encodes heme oxygenase-1 (HO-1), an antioxidant enzyme that participates in the regulation of GPX4 expression (Dang et al., 2022; He et al., 2020).

Nrf2 and its downstream factor HO-1 exhibit both anti-ferroptotic and anti-inflammatory properties. Nrf2 directly regulates the expression of SLC7A11, glutamate-cysteine ligase catalytic subunit (GCLC), glutamate-cysteine ligase modifier subunit (GCLM), and glutathione synthase (GSS), which catalyze GSH synthesis, thereby enhancing GPX4 activity and suppressing ferroptosis (Zhang, 2025). HO-1 is an antioxidant enzyme that also regulates the transcription of GPX4 (Lin et al., 2023; Lv et al., 2024). Moreover, both Nrf2 and HO-1 inhibit the activation of NF-κB, thereby decreasing the levels of proinflammatory cytokines (Bellezza et al., 2012; Yan et al., 2021).

#### 4.3.2 SIRT1/Nfr2/HO-1/GPX4

The activity of Nrf2 signaling is enhanced by SIRT1 via increasing its expression, decreasing the expression of Keap1, and promoting its binding ability to antioxidant response element (ARE) (the promoter region of downstream antioxidant enzymes) (Patel et al., 2022). The SIRT1/Nrf2/HO-1/GPX4 pathway was activated in chronic social defeat stress (CSDS)-induced mice treated with edaravone (EDV), which suppressed neuronal ferroptosis and alleviated depressive-like behaviors. Moreover, NF-κB activation was inhibited by decreasing the protein level of TLR4, resulting in suppression of inflammation. While Nfr2 and HO-1 are recognized inhibitors of NF-κB, this specific mechanism was not discussed in the study (Dang et al., 2022). Besides, acupuncture (AP) also activated the Sirt1/Nrf2/HO-1/GPX4 pathway to alleviate depressive-like behaviors in CUMS-induced rats. Furthermore, AP attenuated NF-κB activation and inhibited the expression of IL-1β and TNF-α but did not reverse the level of TLR4, an effect that might be attributed to the suppressive effects of Nrf2 and HO-1 on NF-κB (Shen et al., 2024a). Another study also reported that activation of Nrf2/HO-1 attenuated neuronal injury and depressive-like behaviors, although the BDNF-extracellular regulated protein kinases (ERK)1/2-cAMP-response element binding protein (CREB) axis also contributed to these neuroprotective effects (Zhang J. B. et al., 2025).

#### 4.3.3 HMGB1/5HT7R/Nrf2/xCT/GPX4

While HMGB1 functions as a DAMP and promotes inflammation, it has also been reported to promote ferroptosis via promoting hepcidin expression and secretion (which blocks iron efflux and increases intracellular iron level) and by suppressing the Nrf2/HO-1 axis (Davaanyam et al., 2023; Guo et al., 2023; Liu et al., 2024; Chen et al., 2022). Serotonin receptor 7 (5-HT7R), a G protein-coupled receptor (GPCR), has been reported to contribute to depression, whereas its blockade exerts antidepressant effects (Bijata et al., 2022; Kim et al., 2016; Crispino et al., 2020). The HMGB1/5HT7R/Nrf2/xCT/GPX4 pathway was found to promote ferroptosis of M2 microglia in transient middle cerebral artery occlusion (tMCAO) mice, which promoted ischemic brain injury and depressive-like behaviors. Following HMGB1 inhibition or 5-HT7R knockout, the downstream Nrf2/xCT/GPX4 signaling pathways was upregulated, resulting in alleviated depressive-like behaviors (Du et al., 2025). By simultaneously suppressing inflammation and ferroptosis, Nrf2 and its upstream regulators represent endogenous brakes on the vicious cycle, and their activation is a promising strategy to reset homeostasis.

## 5 The inflammation-ferroptosis vicious cycle in depression

**Figure 3:**
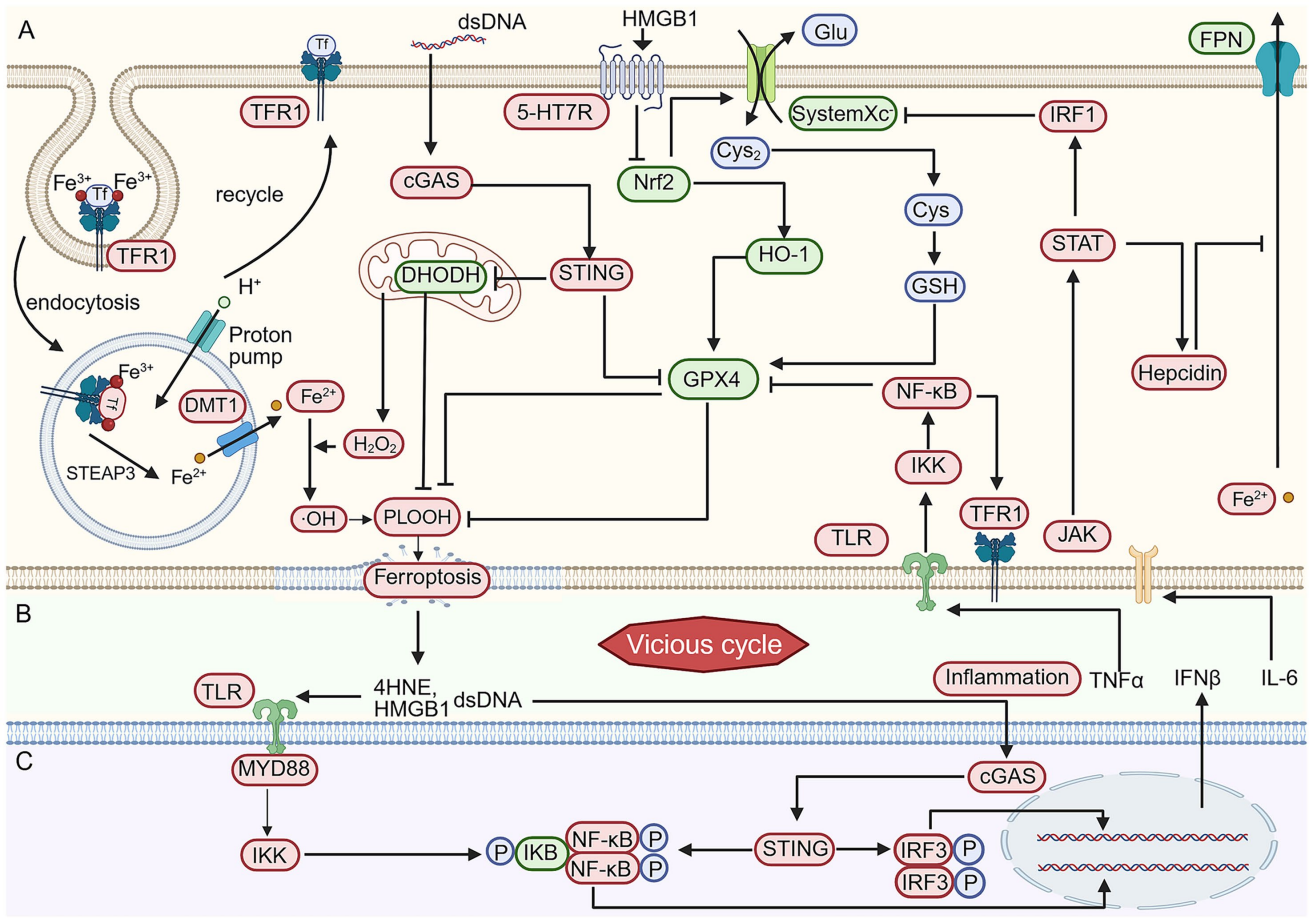
The vicious cycle between ferroptosis and neuroinflammation in depression. **(A)** Iron metabolism and pathways linking inflammation to ferroptosis. On the left, the physiological process of iron transport from circulation into the cell is shown. TFR1 binds transferrin (TF) and internalizes the TF–TFR1 complex via endocytosis. In the acidic endosomal environment, STEAP family reductases facilitate the release of ferrous iron (Fe^2+^), which is then exported into the cytosol by DMT1. The TF–TFR1 complex is subsequently recycled to the plasma membrane. Cytosolic Fe^2+^ reacts with hydrogen peroxide (H_2_O_2_) derived from mitochondria via the Fenton reaction, generating hydroxyl radicals (•OH), which are critical for the production of lipid hydroperoxides (PLOOH). Accumulation of PLOOH ultimately leads to ferroptosis. Two major pathways counteract PLOOH accumulation: the mitochondrial DHODH pathway and the GPX4 pathway. DHODH, a mitochondrial enzyme, primarily scavenges PLOOH within mitochondria. GPX4 relies on the System Xc–GSH axis: System Xc^−^ (composed of SLC7A11 and SLC3A2) imports cystine, which is reduced to cysteine and used for glutathione (GSH) synthesis; GPX4 then utilizes GSH as a cofactor to reduce PLOOH in the plasma membrane. Both pathways are suppressed by the cGAS/STING signaling axis. Specifically, dsDNA activates cGAS, which in turn activates STING; STING inhibits both DHODH and GPX4. Inflammatory cytokines also act via TLRs to activate IKK, which then activates NF-κB. NF-κB promotes TFR1 expression while repressing GPX4 expression. JAK is activated by inflammatory signals, leading to STAT activation, which induces the expression of hepcidin and IRF1. Hepcidin inhibits iron export via FPN, causing cytoplasmic Fe^2+^ accumulation. IRF1 suppresses System Xc^−^ (specifically SLC7A11) expression. The Nrf2/HO-1 axis normally activates the GPX4 pathway. However, under pathological conditions, HMGB1 promotes 5-HT7R expression, and 5-HT7R inhibits Nrf2 expression, thereby promoting ferroptosis. **(B)** Ferroptotic cells release damage-associated molecular patterns (DAMPs) such as 4-HNE, HMGB1, and dsDNA. These DAMPs act on neighboring cells to induce further production of pro-inflammatory cytokines. In turn, these cytokines feedback to promote ferroptosis through the pathways described in **(A)**, thus establishing a vicious cycle from ferroptosis to inflammation and back to ferroptosis. **(C)** Detailed mechanisms of DAMP-induced inflammation following ferroptosis. 4-HNE and HMGB1 bind to TLRs, activating IKK and subsequently NF-κB, leading to the transcription and release of downstream inflammatory cytokines. dsDNA activates the cGAS/STING pathway, which then activates IRF3. IRF3 drives the transcription, synthesis, and release of additional inflammatory cytokines, completing the link from ferroptosis to inflammation. Created in BioRender. yao, L. (2026) https://BioRender.com/dx6ty03

The preceding sections have delineated the independent roles of neuroinflammation and ferroptosis in depression, as well as the molecular pathways that interconnect them. Here, we synthesize these findings into a cohesive model of a bidirectional vicious cycle that amplifies neuropathology and drives the progression of depressive disorders (Figure 3).

Inflammation drives ferroptosis. cGAS/STING activation induces GPX4 degradation and DHODH downregulation in neurons and microglia (You et al., 2025; Zhang J. B. et al., 2025; Woo et al., 2024; Sui et al., 2025). JAK/STAT signaling via IL-6/IFN-γ upregulates hepcidin, blocking iron export, while IRF1 inhibits SLC7A11, reducing glutathione and GPX4 activity (Kowdley et al., 2021; Qian et al., 2014; Xiong et al., 2016; Feng et al., 2025; Kong et al., 2021; Zhou Y. et al., 2024). NF-κB suppresses GPX4 and system Xc^−^, upregulates TFR1, thereby promoting microglial ferroptosis (Zheng et al., 2024; García-Weber and Arrieumerlou, 2021; Xie et al., 2025). Nrf2 counteracts these effects by upregulating SLC7A11, GCLC, and HO-1, enhancing glutathione synthesis and GPX4 activity while inhibiting NF-κB (Zhang, 2025; Lin et al., 2023; Lv et al., 2024; Bellezza et al., 2012; Yan et al., 2021). Ferroptosis fuels neuroinflammation. Ferroptotic cells release DAMPs (4-HNE, HMGB1, and dsDNA), which activate the TLR4-MyD88-NF-κB and cGAS-STING-TBK1-IRF3 pathways in glia, driving the production of IL-1β, IL-6, TNF-α, and type I interferon (Wang et al., 2019; Liu et al., 2015; Feng et al., 2024; Imai et al., 2008; Dvorkin et al., 2024). Iron dyshomeostasis in glial cells—even in the absence of cell death—contributes to the vicious cycle: iron-loaded microglia directly secrete IL-1β and IL-18 to fuel neuroinflammation (Ryan et al., 2023), while reactive astrocytes release hepcidin and pro-inflammatory cytokines, which elevate neuronal iron load and sensitize neurons to ferroptosis (Xiong et al., 2016; Davaanyam et al., 2023; Moro et al., 2025). This glial iron-driven response amplifies both inflammatory and ferroptotic cascades, perpetuating the cycle.

The vicious cycle drives depression pathology. Within this self-amplifying loop, ferroptosis of distinct CNS cell types produces cumulative structural and functional damage that underlines depressive symptoms. Neuronal ferroptosis directly contributes to hippocampal atrophy and neuronal loss in the prefrontal cortex, manifesting as cognitive deficits, impaired spatial learning and memory, and mood dysregulation (Dang et al., 2022; Tian et al., 2023; Ma et al., 2025; Zou et al., 2025; Chen et al., 2024; Bao et al., 2021; Dai et al., 2023). Microglial ferroptosis creates a neurotoxic environment that aggravates lipid peroxidation in neighboring neurons, further amplifying neuronal damage and perpetuating neuroinflammation (Ryan et al., 2023). Astrocyte ferroptosis disrupts iron and inflammatory homeostasis, indirectly promoting neuronal injury through secreted factors (Xiong et al., 2016; Davaanyam et al., 2023; Moro et al., 2025). Together, these cell-type-specific ferroptotic events converge to produce the core pathological features of depression: hippocampal shrinkage, disrupted neurogenesis, and impaired prefrontal cortical function, ultimately driving the clinical manifestations of anhedonia, executive dysfunction, and persistent low mood (Troubat et al., 2021; Reyes-Martínez et al., 2023).

## 6 Therapeutic opportunities targeting the ferroptosis-neuroinflammation axis

As inflammation and ferroptosis are both promoters of depression and their bidirectional interaction may exacerbate the progression of depression, drugs that suppress both inflammation and ferroptosis appear to have potential antidepressant effects. Therefore, agents capable of breaking the vicious cycle by simultaneously suppressing neuroinflammation and ferroptosis represent a rational and promising therapeutic approach. In this section, we review several agents that exhibit both anti-inflammatory and anti-ferroptotic properties and may therefore hold therapeutic potential for depression (Table 1).

**Table 1:** Potential therapy strategies target neuroinflammation and ferroptosis of CNS cells.

Agent	Edaravone	Anacardic acid	Cynaroside	Saikosaponins (SSd, SSa, SSB2)	MicroRNA
Key molecular targets	Anti-ferroptosis: Sirt1-Nfr2-HO-GPX4 Anti-inflammation: TLR4-NF-κB	Anti-ferroptosis: downregulate Tfr1, upregulated GPX4 Anti-inflammation: IKK-NF-κB	Anti-ferroptosis: Nrf2-HO-1 and upregulated SLC7A11 and GPX4 Anti-inflammation: inhibited activation of NF-κB and promotes MI-to-M2 polarization	Anti-ferroptosis: upregulated Nrf2, GPX4 and SLC7A11 Downregulated ACSL4 and TFR1 Anti-inflammation: NLRP3 ubiquitination and HMGB1-TLR4-NF-κB	Anti-inflammation: upregulate miR-27a to suppress SYK/NF-κB axis; miR-207 and miR-182-5p mimics inhibit NLRP3 inflammasome activation
Main effects	Attenuated loss of neurons, microglial activation, oxidative stress and astrocyte dysfunction	Attenuated loss of neurons, neurodegeneration, brain tissue defects and BBB damage	Reduced ferroptosis of microglia, neuronal damage	Inhibited activation of microglia	Reduced pro-inflammatory cytokine release, attenuated neuroinflammation, ameliorated depressive-like behaviors
Used experimental models	Depression models: CSDS Social isolation (SI)	Traumatic brain injury (TBI) model (lacking direct evidence in depression models)	Depression models: CUMS Others: cerebral ischemia–reperfusion (I/R) model *In vitro* neurodegenerative model	Depression models: CUMS LPS-induced models	Depression models: LPS/CSDS-induced rodent models, chronic stress-induced depressive-like behaviors; *in vitro*: LPS + ATP-treated microglia; NK cell-derived exosomes in depressed mice
Advantages	Multitarget, FDA-approved,	Natural, multitarget Metal chelation ability	Polarized M1 microglia to M2 Good neuroprotective data	Diverse types with various mechanisms	precise modulation of inflammatory pathways, potential for multi-target regulation, versatile delivery strategies (e.g., exosome-mediated)
Limitations	No benefit in alleviating oxidative DNA	Limited data in depression and lower efficacy of anti-ferroptosis than Fer-1	Relatively few studies focused on depression	Poor bioavailability	Direct evidence for regulation of ferroptosis-related pathways in depression remains largely unexplored

### 6.1 Edaravone

EDV, an anti-oxidant and FDA-approved drug, has been reported to have anti-inflammatory and neuroprotective properties. In addition, it was reported to produce antidepressant effects in a CSDS-induced mouse model of depression. EDV significantly attenuated the loss of neurons in the hippocampus and medial prefrontal cortex (mPFC), microglial activation, oxidative stress damage, and astrocyte dysfunction via the Sirt1-Nfr2-HO-GPX4 pathway. As GPX4 plays a critical role in preventing ferroptosis, the CSDS-induced downregulation and EDV-induced upregulation of GPX4 suggest that ferroptosis contributes to depression development. On the other hand, the increased levels of proinflammatory cytokines such as IL-1β, IL-6, and TNF-α induced by CSDS were reversed by EDV treatment through downregulation of the TLR4-NF-κB pathway (Dang et al., 2022). Another study also reported that EDV alleviated depressive-like behaviors by reversing the levels of GSH (Montazeri et al., 2023). However, EDV was not found to confer benefit in alleviating oxidative DNA damage (Herbet et al., 2019).

On the other hand, EDV upregulates the expression of genes involved in neurotransmitter turnover, adenosine A1 receptor (Adora1), solute carrier family 6 member 15 (Slc6a15), catechol-O-methyltransferase (Comt). In addition, EDV regulates the hypothalamic–pituitary–adrenal (HPA) axis via downregulation of FK506 binding protein 5 (FKBP5), a protein involved in glucocorticoid receptor (GR) trafficking to the nucleus, which may contribute to normalization of stress-induced abnormalities in the HPA axis (Herbet et al., 2019).

The multi-target capability of EDV enables it to target and inhibit neuroinflammation and ferroptosis of nerve cells simultaneously. Its action on the Sirt1-Nfr2-HO-GPX4 pathway directly exerts antioxidant effects, while suppression of the TLR4-NF-κB pathway dampens neuroinflammation signaling. This dual action perfectly disrupts the inflammation-ferroptosis vicious cycle and thus makes EDV a promising antidepressant. However, its lack of effect on oxidative DNA damage suggests that combination therapy with other agents may be necessary. By targeting both arms of the vicious cycle—Sirt1/Nrf2/HO-1/GPX4 for ferroptosis and TLR4/NF-κB for inflammation—EDV exemplifies a cycle-disrupting therapeutic strategy.

### 6.2 Anacardic acid

Anacardic acid (AA) is found in the agro waste from cashew nut processing, cashew nut shell liquid (CNSL), positioning it an alternative green source. As an antioxidant, AA inhibits various enzymes involved in the production of ROS and exhibits high selectivity toward Fe^2+^ and Cu^2+^ ions in metal chelation (Hamad and Mubofu, 2015). Moreover, AA suppresses inflammation via blocking the NF-κB pathway by inhibiting inhibitor of kappa B kinase (IKK) activation (Hemshekhar et al., 2012). AA has been reported to inhibit ferroptosis through downregulation of TfR1 and upregulation of GPX4; however, the anti-ferroptotic effect of AA was less effective than Fer-1, and suppress inflammation by downregulating the level of IL-6, TNF-α, IL-1, and CXCL1, thereby attenuated traumatic brain injury (TBI) -induced neuronal loss, neurodegeneration, brain tissue defects, and BBB damage (Liu Y. et al., 2023). However, while one study reported an antidepressant effect of AA mediated by the L-arginine–nitric oxide–serotonergic system, its anti-inflammatory and anti-ferroptotic effects have not been investigated in the context of depression (Júnior et al., 2019).

AA, a natural iron chelator, not only inhibits inflammation by suppressing the NF-κB pathway but also directly modulates ferroptosis cascade via downregulating TfR1 and upregulating GPX4. However, although its antidepressant effects have been demonstrated through other mechanisms, its anti-inflammatory and anti-ferroptotic properties remain to be validated in depressive models. Further studies are needed to confirm whether these effects contribute to its antidepressant potential. Given its ability to chelate iron and suppress NF-κB, AA may interrupt the vicious cycle at two distinct nodes, warranting further investigation in depressive models.

### 6.3 Cynaroside

Cynaroside (Cyn), also known as luteoloside and cynaroside, is an extract derived from the herb honeysuckle with antioxidant and anti-inflammatory properties. Cyn was reported to upregulate anti-ferroptotic genes HO-1, SLC7A11, and GPX4 in LPS-induced BV-2 cells (microglia from mice), thereby reversing the levels of MDA, GSH and Fe^2+^, and thus suppressing the ferroptosis of microglia. Moreover, the levels of the M1 microglia marker CD16/32 and proinflammatory cytokines IL-6 and TNF-α were downregulated while the levels of anti-inflammatory cytokines IL-4 and IL-10 were upregulated. Furthermore, Cyn inhibits the activation of NF-κB and microglia, thereby downregulating the proinflammatory cytokines in the hippocampus and reducing neuronal damage in CUMS-induced mice. In addition, the effects of Cyn on alleviating depressive-like behaviors are similar to those of fluoxetine (FLX) (Zhou Y. et al., 2024).

Studies that are associated with the effects of Cyn on depression are remain limited. However, we did find its anti-inflammatory and anti-ferroptosis effects in other studies related to CNS diseases. For example, in one study that used middle cerebral artery occlusion (MCAO) to explore the effects of Cyn in cerebral ischemia–reperfusion (I/R), Cyn induced the activation of Nrf2 and peroxisome proliferative activated receptor γ (PPARγ), contributing to the inhibition of the NF-κB inflammatory pathway and potentially exerting antioxidant effects, thereby attenuating neuroinflammation and cerebral infarction (Li et al., 2019). Another study investigated the neuroprotective effects of Cyn in an *in vitro* neurodegenerative model (SH-SY5Y neuroblastoma cells induced with 6-hydroxydopamine hydrobromide [6-OHDA]) and reported that Cyn suppressed 6-OHDA-induced mitochondrial and nuclear damage, although the levels of ROS were not affected. Moreover, Cyn was found to decrease the levels of TNF-α and increase the levels of IL-10 in RAW264.7 monocytes/macrophages, suggesting its anti-inflammatory properties (Rehfeldt et al., 2022).

Cyn emerges as a promising candidate, as it not only polarizes microglia from a pro-inflammatory M1 phenotype to the anti-inflammatory M2 phenotype—a key switch for resolving neuroinflammation—but also reverses ferroptosis markers such as GSH, GPX4, and MDA. Furthermore, it alleviates depressive-like behaviors and exhibits neuroprotective properties in other CNS diseases. Its dual action—promoting microglial polarization toward an anti-inflammatory phenotype and reversing ferroptosis markers—positions Cyn as a particularly attractive cycle-modulating agent.

### 6.4 Saikosaponins

Saikosaponins, extracted from medicinal plants such as Radix Bupleuri, belong to the triterpenoid saponin family. Saikosaponins have been reported to have antidepressant, anti-ferroptotic, and anti-inflammatory properties (Li et al., 2018). Several saikosaponin subtypes have been identified, including a, b, c, d, m, n, p, and t (Wong et al., 2009). Saikosaponins inhibit neuroinflammation via various pathways. For example, saikosaponin d (SSd) regulates NLRP3 ubiquitination through the E3 ubiquitin ligase membrane associated ring-CH-type finger 7 (MARCHF7) to reduce the levels of NLRP3 inflammasome-dependent inflammatory cytokines IL-1β and IL-18, thereby alleviating depressive-like behaviors CUMS-induced mice (Gao et al., 2024). In addition, SSd downregulates the expression of NF-κB, upregulating fibroblast growth factor 2 (FGF2), a neurotrophic growth factor involved in regulating synaptic plasticity and neuronal growth (Chao et al., 2020). Furthermore, another study reported that SSd inhibited the activation of the HMGB1 and TLR4/NF-κB pathways to reduce neuroinflammation (Su et al., 2020). Saikosaponin a (SSa) and saikosaponin B2 (SSB2) also have the ability to downregulate the levels of NF-κB, contributing to the inhibition of neuroinflammation (Wang et al., 2023; Tan et al., 2025). In detail, SSa downregulates TLR4 and NF-κB while BDNF is upregulated, promoting the alleviation of depressive-like behaviors in CUMS-induced mice (Tan et al., 2025). The TLR4-NF-κB pathway is also inhibited by SSB2 in LPS-induced microglia, which suppresses the activation of microglia and reduces the levels of IL-1β, IL-6, and TNF-α, increasing the anti-inflammatory cytokine IL-10 (Wang et al., 2023).

Saikosaponins, particularly, SSB2 and SSA, are antioxidants that may contribute to the suppression of ferroptosis (Wang et al., 2023; Tan et al., 2025). SSB2 upregulates the expression of Nrf2 and SLC7A11, thereby increasing the levels of GSH. Moreover, the central anti-ferroptotic protein, GPX4, a central anti-ferroptotic protein, was also upregulated by SSB2. On the other hand, ACSL4 and TFR1 are downregulated, which inhibits lipid peroxidation and decreases the levels of intracellular iron. Notably, the anti-inflammatory and anti-ferroptotic effects of SSB2 were found to be GPX4-dependent (Wang et al., 2023). SSA also exhibits anti-ferroptotic potential, attenuating the levels of ROS and MDA while the levels of GSH and SOD were upregulated (Tan et al., 2025).

The saikosaponin family exerts its antidepressant effects through various pathways: promoting ubiquitination of NLRP3 inflammasome, inhibiting NF-κB pathway, upregulating the expression of anti-ferroptotic proteins (GPX4, Nrf2, and SLC7A11) and attenuating lipid peroxidation. SSd appears to primarily target inflammation, while SSA and SSB2 target both inflammation and ferroptosis. This structural diversity within the saikosaponin family offers a rich chemical landscape for antidepressant drug development. Future studies should further compare the antidepressant efficacy of different saikosaponins and optimize their brain delivery to translate this ancient herbal knowledge into modern therapeutic agents. Their ability to interrupt distinct segments of the vicious cycle—NLRP3 inflammasome, NF-κB, and Nrf2/GPX4—provides a multi-target strategy for dismantling the cycle.

### 6.5 MicroRNA

MicroRNAs (miRNAs) are small non-coding RNAs that function as sequence-specific guides within the RNA-induced silencing complex (RISC) to post-transcriptionally regulate gene expression, predominantly by decreasing target mRNA levels or repressing their translation (Mohr and Mott, 2015; Saliminejad et al., 2019). Current research on microRNA (miRNA)-based therapeutic strategies for depression remains predominantly at the preclinical stage, with evidence primarily derived from two complementary approaches. One line of investigation involves the pharmacological modulation of endogenous miRNA expression to influence inflammatory pathways. For instance, the flavonoid isoliquiritin has been shown to upregulate miR-27a, thereby suppressing inflammatory signaling, reducing hippocampal levels of pro-inflammatory cytokines, and ultimately ameliorating depressive-like behaviors in rodent models (Li et al., 2021). Another strategy entails the direct administration of synthetic miRNA mimics to restore or enhance protective signaling pathways. For example, exosomes from unstressed mouse NK cells can suppress the release of pro-inflammatory cytokines from stressed astrocytes *in vitro*, whereas direct intracranial administration of a miR-207 mimic recapitulates the overall antidepressant phenotype in depressed mice (Li D. et al., 2020). Furthermore, supplementation with miR-182-5p mimics has been shown to inhibit hippocampal NLRP3 inflammasome activation, thereby attenuating neuroinflammation and mitigating chronic stress-induced depressive-like behaviors (Li et al., 2021).

While the anti-inflammatory roles of miRNAs in depression are well-documented, their involvement in regulating ferroptosis-related pathways—such as GPX4, SLC7A11, and ACSL4—remains largely unexplored. Given the critical intersection between neuroinflammation and ferroptosis in depression, future studies investigating miRNA-mediated regulation of ferroptosis may reveal novel therapeutic opportunities to disrupt this vicious cycle.

## 7 Discussion

Among these compounds, EDV and saikosaponins exhibit the most robust multi-target mechanisms and have demonstrated direct efficacy in depression models. Cyn exhibits a unique action in targeting microglial polarization toward an anti-inflammatory phenotype but requires more validation in depression contexts. AA remains the most preliminary in depression research, despite being a potential multi-target agent with anti-inflammatory and anti-ferroptotic properties. Besides, for natural compounds like saikosaponins and cynaroside, pharmacokinetics are the major concerns that must be addressed through formulation engineering or delivery strategies. Although saikosaponins have poor intestinal absorption due to their large molecular mass, high molecular flexibility, and high hydrogen-bonding capacity—features that limit membrane permeability—certain subtypes (saikosaponin a, b1, d, g, c) have been shown to exhibit relatively high blood–brain barrier permeability in a microdialysis experiment, making them potential antidepressants (Yu et al., 2012; Gao et al., 2020). In contrast, while pharmacokinetic studies have characterized the rapid plasma clearance of cynaroside after intravenous administration, its oral bioavailability and ability to cross the blood–brain barrier remain poorly understood (Huang et al., 2014). Nevertheless, its antidepressant effects have been demonstrated in mice via abdominal injection, suggesting that its pharmacokinetic properties—particularly regarding absorption and brain distribution—deserve further investigation (Zhou Y. et al., 2024). In summary, simultaneously targeting neuroinflammation and ferroptosis pathways is a feasible strategy, offering new hope for patients with treatment-resistant depression. Central to this strategy is the recognition that neuroinflammation and ferroptosis are not independent events but are coupled in a self-perpetuating vicious cycle. Breaking this cycle at either its inflammatory or ferroptotic arm—or ideally both—holds the potential to restore neural homeostasis and alleviate depressive symptoms.
